# *CLOCK* Gene Variation Is Associated with the Incidence of Metabolic Syndrome Modulated by Monounsaturated Fatty Acids

**DOI:** 10.3390/jpm11050412

**Published:** 2021-05-14

**Authors:** Dayeon Shin, Kyung-Won Lee

**Affiliations:** 1 Department of Food and Nutrition, Inha University, Incheon 22212, Korea; dyshin@inha.ac.kr; 2 Department of Home Economics Education, Korea National University of Education, Cheongju 28173, Korea

**Keywords:** *CLOCK* polymorphisms, metabolic syndrome, dietary monounsaturated fatty acids, Korean Genome and Epidemiology Study (KoGES)

## Abstract

The circadian locomotor output cycles kaput (*CLOCK*) gene plays a crucial role in regulating circadian rhythms through its transcription factor gene product. The objective of this study was to investigate the association between *CLOCK* rs1801260 and the incidence of metabolic syndrome modulated by dietary monounsaturated fatty acid (MUFA) intake in Korean adults. Using a dataset from the Ansan-Ansung Cohort Study of the Korean Genome and Epidemiology Study, 3608 Korean adults were included after an average of nine years of follow-up. Men who were minor allele carriers (G allele) of *CLOCK* rs1801260 had a 18% higher incidence of metabolic syndrome than non-carriers [hazard ratio (HR), 1.18; 95% confidence interval (CI), 1.00–1.40; *p* Value = 0.047]. By dichotomizing dietary MUFA intake, we observed that men who were minor allele carriers (G allele) of *CLOCK* rs1801260 had a 42% increased incidence of metabolic syndrome when dietary MUFA intake was ≤3.5% (HR: 1.42, 95% CI 1.23–1.81; *p* Value = 0.004). No significant association was found between *CLOCK* rs1801260 and the incidence of metabolic syndrome modulated by dietary MUFA intake in women. *CLOCK* polymorphisms affected metabolic syndrome, modulated by dietary MUFA intake in men. These results suggest the significance of *CLOCK* genes in the pathogenesis of metabolic syndrome and the modulating role of dietary MUFA intake and provide new insights into the underlying mechanisms connecting the circadian system, dietary factors, and metabolic syndrome.

## 1. Introduction

The transcription factor encoded by the circadian locomotor output cycles kaput (*CLOCK*) gene is a crucial element of the molecular circadian clock [1]. Energy balance is influenced by this transcription factor, thus impacting metabolic pathways [2]. A large body of evidence suggests that genetic variation in *CLOCK* is associated with the development of metabolic syndrome and its components [1,3,4]. Mutations in *CLOCK* genes may be a causal factor for the expression of metabolic syndrome components by altering transcriptional regulation, with *CLOCK* mutant mice showing impaired glucose tolerance [5]. Disruption of *CLOCK* genes can lead to impaired glucose tolerance and diabetes in mouse models [6].

Metabolic syndrome is affected by both genetic and dietary factors. *CLOCK* genetic polymorphisms have been associated with metabolic syndrome, and one of the most studied *CLOCK* gene polymorphisms at the 3′-untranslated region is rs1801260 [7]. Dietary factors play a key role in the development of metabolic syndrome. Consumption of dietary monounsaturated fatty acids (MUFA) promotes improved metabolic profiles such as improving insulin sensitivity, maintaining healthy blood lipid profiles and regulating blood glucose levels [8]. However, studies evaluating how *CLOCK* genetic polymorphisms predispose an individual to metabolic syndrome, and how this relationship is modulated by MUFA, are lacking.

Research investigating the relationship between *CLOCK* single nucleotide polymorphisms (SNPs) and the incidence of metabolic syndrome modulated by MUFA intake using a prospective cohort study design is limited, and the role of *CLOCK* SNPs in metabolic syndrome in Korean adults is unclear. Thus, the primary objective of this study was to investigate the association between the common *CLOCK* SNP rs1801260 and the incidence of metabolic syndrome in Korean adults. Additionally, we assessed the association of *CLOCK* SNP rs1801260 with the incidence of metabolic syndrome modulated by MUFA intake.

## 2. Material and Methods

### 2.1. Study Design and Participants

Data from the Ansan-Ansung Cohort Study of the Korean Genome and Epidemiology Study (KoGES), which is an ongoing prospective study conducted by the Korea National Institute of Health, was used for this study [9]. Briefly, the Ansan-Ansung community-based cohort study began in 2001–2002 to examine the dietary and lifestyle factors that affect the incidence and prevalence of chronic diseases in the Korean population. A total of 10,030 adults, aged 40–69 years, who resided in Ansan (urban) and Ansung (rural) were recruited. The participants were followed up bi-annually, and we utilized the follow-up data until 2016.

From the 10,030 participants at baseline examination (2001–2002), participants who did not follow up at least once during the follow-up (*n* = 912), had no information on metabolic syndrome at baseline (*n* = 9), had a diagnosis of metabolic syndrome at baseline (*n* = 2800), had a diagnosis of cancer (*n* = 156), had no information on dietary information (*n* = 189), had implausible energy intake (<500 kcal/day or >5000 kcal/day; *n* = 52), had missing information on confounding variables (*n* = 74), and had no information on SNP rs1801260 (*n* = 2230) were excluded, and the final analytic sample was 3608 (1839 men and 1769 women). The study protocol was reviewed and approved by the Institutional Review Board (IRB) of Inha University on 31 January 2020 (IRB No. 2001291A).

### 2.2. Dietary Assessment

Dietary data were obtained by well-trained interviewers using a 103-item semi-quantitative food frequency questionnaire (FFQ) at baseline. In order to assess the usual dietary intake of the Korean adults who participated in the KoGES, this validated FFQ was developed and used [10]. All study participants were asked how often they consumed each food item during the previous 12 months. A total of nine frequency responses, ranging from never or almost never to ≥3 times per day, were collected [10]. The usual intake of foods and nutrients, including MUFA, was calculated by multiplying the frequency of consumption of each food item and nutrient contents for its corresponding food item utilizing a nutrient database (CAN-Pro 2.0) developed by the Korean Nutrition Society [11]. In the present study, MUFA were assessed from FFQ as a percentage contribution from energy (% energy).

### 2.3. Assessment of Metabolic Syndrome

Diagnosis of metabolic syndrome was carried out according to guidelines of the National Cholesterol Education Program Adult Treatment Panel III [12] and the International Diabetes Federation [13]. New-onset metabolic syndrome was identified based on the presence of three or more of the following conditions: (1) abdominal obesity (waist circumference ≥ 90 cm for men and ≥80 cm for women); (2) elevated blood pressure (systolic blood pressure ≥ 130 mmHg or diastolic blood pressure ≥85 mmHg or antihypertensive drug use or antihypertensive treatment); (3) elevated fasting blood glucose (FBG) ≥ 100 mg/dL or insulin or oral drug therapy; (4) elevated triglyceride levels (fasting triglyceride ≥ 150 mg/dL); and (5) low high-density lipoprotein (HDL) cholesterol levels (fasting HDL-cholesterol < 40 mg/dL for men and <50 mg/dL for women). In the survival analysis, survival time for individuals who reported any diagnosis of metabolic syndrome during the follow-up period was defined as the time between the baseline and the date of the new onset of metabolic syndrome. Participants who did not acquire metabolic syndrome were censored at the last date of follow-up examination. Survival time was calculated as the time difference between the baseline and the last follow-up.

### 2.4. Genotyping and Imputation

Genomic information was obtained from participants’ DNA samples that were isolated from their peripheral blood samples. Genetic data were collected utilizing the Korean Biobank Array (Korean Chip, K-CHIP) through the K-CHIP consortium [14]. K-CHIP, including approximately 833,535 SNPs specific to the Korean population, was designed by the Center for Genome Science, Korea National Institute of Health [14]. For standard quality control procedures, *p* Value for Hardy–Weinberg equilibrium ≥ 1.0 × 10^−6^ and call rate ≥ 95% were used [14]. Genetic data were imputed using Shapeit v2 and IMPUTE v2, with the 1,000 Genomes Project phase 3 reference [14].

### 2.5. Statistical Analyses

Genetic analysis was performed using PLINK (version 1.90 beta, https://www.cog-genomics.org/plink/1.9). All analyses were performed separately for men and women. Study participants were categorized into two groups, according to the median value of the percentage of energy acquired from MUFA intake for men and women, respectively. Baseline sociodemographic, clinical, and lifestyle characteristics of the study participants were computed using Chi-square tests for categorical variables and one-way analysis of variance (ANOVA) for continuous variables. Using multivariable Cox proportional hazards models, hazard ratios (HRs) and 95% confidence intervals (CIs) for the incidence of metabolic syndrome were estimated using the *CLOCK* SNP rs1801260 under a dominant genetic model stratified by MUFA intake. The potential covariates that were entered into the model were age (years), area of residence (Ansung, Ansan), education level [elementary school or lower (<7 years of school completed), middle/high school (7–12 years), college or higher (>12 years)], smoking status (never, former smoker, current smoker), alcohol consumption (g/day), physical activity [metabolic equivalent task (MET)-h/week], body mass index (BMI; kg/m^2^), and family history of diabetes (determined on the basis of self-reports: yes, no). All statistical analyses were performed using SAS software (version 9.4; SAS Institute, Cary, NC, USA). A two-sided *p* Value of < 0.05, was considered statistically significant.

## 3. Results

Briefly, 1658 of the study population had an incidence of metabolic syndrome during an average of nine years of follow-up. Table 1 presents the sociodemographic, clinical, lifestyle, and genetic characteristics of the study participants at baseline. The characteristics of age, insulin level, glucose level, insulin resistance, triglyceride level, HDL cholesterol level, waist circumference, systolic blood pressure, diastolic blood pressure, BMI, alcohol intake, MET-h/week, region, smoking status, and dietary fat composition all significantly differed by sex (all *p* Values < 0.0001). 

The sociodemographic, clinical, and lifestyle characteristics of the study participants at baseline according to the *CLOCK* rs1801260 genotype are presented in Table 2. No statistically significant differences were detected in age, insulin level, glucose level, insulin resistance [homeostatic model assessment of insulin resistance (HOMA-IR)], triglyceride level, HDL-cholesterol level, waist circumference, systolic and diastolic blood pressure, MET-h/week, region, smoking status, and dietary fat composition between the *CLOCK* rs1801260 genotypes (AA vs. AG + GG) in men and women, respectively. Men with AA rs1801260 genotype had marginally and significantly higher MET-h/week than those with the AG or GG rs1801260 genotype (*p* = 0.06).

Table 3 shows the sociodemographic, clinical, and lifestyle characteristics of the study participants at baseline according to the *CLOCK* rs1801260 genotype and dietary MUFA intake. Dietary MUFA intake was divided by median values: 3.5% of total energy for men and 3% of total energy for women. In men, age, HDL-cholesterol, systolic blood pressure, BMI, alcohol intake, and MET-h/week significantly differed across the rs1801260 genotypes and dietary MUFA intake groups (rs1801260 genotype AA and MUFA ≤ 3.5% of total energy, rs1801260 genotype AA and MUFA > 3.5% of total energy, rs1801260 genotype AG + GG and MUFA ≤ 3.5% of total energy, and rs1801260 genotype AG + GG and MUFA > 3.5% of total energy) (all *p* Values < 0.05). In women, age, glucose level, waist circumference, systolic and diastolic blood pressure, alcohol intake, and MET-h/week all significantly differed across the rs1801260 genotypes and dietary MUFA intake groups (all *p* Values < 0.05). 

The association between *CLOCK* rs1801260 polymorphisms and the incidence of metabolic syndrome is presented in Table 4. Men who were G carriers (AG + GG) showed a significantly higher incidence of metabolic syndrome compared to AA homozygous subjects (HR, 1.18; 95% CI, 1.00–1.40; *p* Value = 0.047) after controlling for age, region, smoking, alcohol consumption, physical activity, family history of type 2 diabetes, and BMI. There was no significant association between the rs1801260 polymorphism and the incidence of metabolic syndrome in women.

We analyzed the modulation of the *CLOCK* rs1801260 polymorphisms and the incidence of metabolic syndrome stratified by dietary MUFA intake (Table 5). We observed that men who were G carriers (AG + GG) with a MUFA intake ≤ 3.5% of total energy had a significantly higher incidence of metabolic syndrome than men with the AA genotype and MUFA intake > 3.5% of total energy (HR, 1.42; 95% CI, 1.23–1.81; *p* Value = 0.004) after adjustment for covariates. No significant associations between *CLOCK* rs1801260 polymorphisms, dietary MUFA intake, and the incidence of metabolic syndrome were observed in women.

## 4. Discussion

In this prospective cohort study, we observed an association between the minor allele carriers of the G allele (AG + GG) of *CLOCK* rs1801260 and the incidence of metabolic syndrome in Korean men. Previous findings have reported that *CLOCK* polymorphisms are associated with metabolic-related biomarkers. Subjects with the major allele had higher insulin sensitivity, lower insulin resistance and lower plasma insulin level compared with subjects who were carriers of the minor allele [3]. Similarly, in a study in which the association of *CLOCK* polymorphisms with metabolic syndrome was investigated in 1100 participants in the Genetics of Lipid Lowering Drugs and Diet Network study, GG carriers of rs1801260 in the study population had significantly increased BMI, systolic blood pressure, fasting insulin level, and HOMA-IR compared with those with AG or AA genotype [15]. Consistent with previous findings, when the saturated fatty acid (SFA) intake was ≥11.8%, subjects with the rs1801260 GG genotype had significantly higher waist circumference than subjects with the AG or AA genotype [15]. In a cohort study of 356 elderly subjects, elderly individuals with the rs1801260 GG genotype had significantly higher fasting glucose levels than those with the AA or AG genotype [16]. *CLOCK* rs1801260 was associated with chronotype [17,18], while other studies did not support this association [19,20]. In a population-based sample of 410 normal middle-aged adults, subjects with the G allele of rs1801260 preferred “eveningness” over “morningness” [17]. In middle-aged Korean adults, the evening chronotype was associated with a higher incidence of diabetes and lower lean mass in men [21]. Previous findings suggest that the evening type is associated with a greater risk for sleep curtailment, and a growing body of evidence suggests that poor-quality sleep and sleep curtailment are associated with an increased risk of obesity [22,23,24].

We hypothesized that *CLOCK* genetic polymorphisms are associated with the incidence of metabolic syndrome modulated by dietary MUFA intake. When stratified by dietary MUFA intake, we found that men with carriers of the minor G allele of rs1801260 (AG + GG) and MUFA ≤ 3.5% of total energy had a higher incidence of metabolic syndrome than those with the major allele of rs1801260 (AA) and MUFA > 3.5%. No significant relationship was found for MUFA > 3.5% of the total energy. Garaulet et al. [15] reported that when MUFA intake was ≥13.2% of the total energy, carriers of the G allele (GG + GC) of rs4580704 had significantly lower plasma glucose concentration and HOMA-IR than non-carriers. When SFA intake was ≥11.8%, minor allele carriers had a larger waist circumference than non-carriers. A protective effect of MUFA on metabolic syndrome, lipid profile, and blood pressure has been reported [8,25,26]. MUFA-rich diets are associated with the inhibition of low-density lipoprotein particle oxidation [27].

Furthermore, dietary MUFA promotes peroxisome proliferator-activated receptor alpha, which enhances fatty acid oxidation and inhibits lipogenesis by suppressing sterol regulatory element binding protein, which, in turn, lowers triglyceride levels in blood [28]. Consistent with these findings, in this study, an increasing incidence of metabolic syndrome was found in men with low intake of MUFA and carrying a minor allele of rs1801260. In contrast, no such significant association could be found in women. Further, women with G minor alleles of rs1801260 did not show increased incidence of metabolic syndrome. The lack of associations in women may be partially due to masking effect by other strong risk factors such as decreased estradiol and estrone level in women who were in postmenopausal stage [29] in the present study. Future studies are warranted to elucidate the potential role of sex on the association *CLOCK* gene polymorphisms and the incidence of metabolic syndrome.

*CLOCK* rs1801260 polymorphisms have been suggested to be associated with obesity [4,15,30,31,32,33], depression [16] and Parkinson’s disease [34]. Among obese patients, individuals with the G minor alleles of rs1801260 (GG + AG) showed higher BMI values compared with those carrying the major allele (AA), and obese patients with the minor allele of rs1801260 were resistant to losing weight compared to those with the major allele [31]. This suggests that the *CLOCK* rs1801260 polymorphism is associated with body weight reduction. In school-age girls with the G allele, carriers of rs1801260 showed increased BMI z-scores at baseline and follow-up [30]. Elderly subjects with the minor allele (rs1801260 GG type) had lower scores on the depression geriatric scale, predisposing greater risk for depression [16]. In a Chinese population, *CLOCK* rs1801260 polymorphism was associated with an increased risk of Parkinson’s disease [34].

This study has several limitations and strengths. The limitation was that this study was conducted on a Korean population, and this finding may not be applicable to other race/ethnicities or populations. Replication studies are warranted to confirm the findings of this study. Despite this limitation, this study has several strengths. To the best of our knowledge, this is the first study to investigate the effect of *CLOCK* polymorphisms on the incidence of metabolic syndrome modulated by dietary MUFA intake in Korean adults. Second, the study utilized a prospective cohort study design to examine the cause–effect relationship of *CLOCK* polymorphisms and the incidence of metabolic syndrome modulated by dietary MUFA intake. Lastly, numerous important confounders, such as physical activity, smoking, drinking habits, and BMI were controlled in the present study.

In conclusion, we found an association between the presence of the minor alleles (AG + GG) of *CLOCK* rs1801260 and low dietary MUFA intake with the incidence of metabolic syndrome in Korean men. The incidence of metabolic syndrome was increased in men who were G carriers (AG + GG) by 42% after adjustment for confounders. These results suggest the significance of *CLOCK* genes in metabolic syndrome risk and the modulating role of dietary MUFA intake. This provides new insights into the underlying mechanisms connecting the circadian system, dietary factors, and metabolic syndrome.

## Figures and Tables

**Table 1:** Sociodemographic, clinical, lifestyle, and genetic characteristics of the study participants at baseline.

	Total (*n* = 3608)	Men (*n* = 1839)	Women (*n* = 1769)	*p* Value ^1^
Age (years)	50.9 ± 8.7	51.4 ± 8.8	50.4 ± 8.6	<0.0001
Insulin (µIU/mL)	7 ± 4.3	6.6 ± 3.9	7.5 ± 4.7	<0.0001
Glucose (mg/dL)	84.3 ± 15.6	86.8 ± 17.1	81.8 ± 13.3	<0.0001
HOMA-IR	1.5 ± 1.2	1.4 ± 0.9	1.5 ± 1.4	<0.0001
Triglyceride (mg/dL)	134.8 ± 82	151.2 ± 99.1	117.7 ± 54.2	<0.0001
HDL-Cholesterol (mg/dL)	47 ± 10.1	45.7 ± 9.9	48.3 ± 10.2	<0.0001
Waist circumference (cm)	79.7 ± 7.6	81.4 ± 6.7	77.9 ± 8.1	<0.0001
Systolic blood pressure (mmHg)	116.6 ± 16.6	118.6 ± 16.2	114.6 ± 16.8	<0.0001
Diastolic blood pressure (mmHg)	77.5 ± 10.6	79.6 ± 10.5	75.3 ± 10.4	<0.0001
BMI (kg/m^2^)	23.7 ± 2.8	23.5 ± 2.6	24 ± 2.9	<0.0001
Alcohol intake (g/day)	9.7 ± 21.4	17.9 ± 27.2	1.3 ± 4.6	<0.0001
MET-h/week	163.7 ± 101.8	172 ± 105.3	155.1 ± 97.4	<0.0001
Region (%)				
Ansung	1495 (41.4%)	712 (38.7%)	783 (44.3%)	0.0007
Ansan	2113 (58.6%)	1127 (61.3%)	986 (55.7%)	
Smoking (%)				
None	2092 (58.0%)	381 (20.7%)	1711 (96.7%)	<0.0001
Past	596 (16.5%)	581 (31.6%)	15 (0.9%)	
Current	920 (25.5%)	877 (47.7%)	43 (2.4%)	
Dietary fat composition				
Total fat (% of energy)	13.3 ± 5	13.9 ± 4.9	12.7 ± 5	<0.0001
SFA (% of energy)	4.4 ± 2	4.6 ± 1.9	4.1 ± 2.1	<0.0001
MUFA (% of energy)	3.5 ± 1.7	3.7 ± 1.7	3.3 ± 1.7	<0.0001
PUFA (% of energy	2.2 ± 0.8	2.2 ± 0.8	2.2 ± 0.8	0.0045
Total fat (g/day)	30.6 ± 17	33.1 ± 17.3	28 ± 16.1	<0.0001
SFA (g/day)	10 ± 6.1	10.9 ± 6.2	9 ± 5.8	<0.0001
MUFA (g/day)	8.1 ± 5.4	8.9 ± 5.6	7.3 ± 5	<0.0001
PUFA (g/day)	5.1 ± 2.8	5.3 ± 2.9	4.8 ± 2.7	<0.0001
*CLOCK* rs1801260 genotype				
AA	2909 (80.6%)	1484 (80.7%)	1425 (80.6%)	0.9583
AG	666 (18.5%)	339 (18.4%)	327 (18.5%)	
GG	33 (0.9%)	16 (0.9%)	17 (0.9%)	

HOMA-IR, homeostatic model assessment of insulin resistance; HDL, high-density lipoprotein; MET, metabolic equivalent task; SFA, saturated fatty acid; MUFA, monounsaturated fatty acid; PUFA, polyunsaturated fatty acid. ^1^ *p* Values based on *t*-tests for continuous variables and Chi-square tests for categorical variables.

**Table 2:** Sociodemographic, clinical, and lifestyle characteristics of the study participants at baseline according to the *CLOCK* rs1801260 genotype.

	Men (*n* = 1839)					Women (*n* = 1769)				
	*CLOCK* rs1801260					*CLOCK* rs1801260				
	AA (*n* = 1484)		AG + GG (*n* = 355)			AA (*n* = 1425)		AG + GG (*n* = 344)		
	Mean	SD	Mean	SD	*p* Value ^1^	Mean	SD	Mean	SD	*p* Value ^1^
Age (years)	50.7	8.4	50.3	8.2	0.49	50	8.2	49.7	8.3	0.62
Insulin (µIU/mL)	6.6	4.1	6.4	3	0.34	7.3	4.5	7.6	3.5	0.19
Glucose (mg/dL)	87.2	17.2	87.7	19.8	0.66	81.4	11.3	82.3	11.4	0.21
HOMA-IR	1.4	0.9	1.4	0.7	0.58	1.5	0.9	1.6	0.8	0.09
Triglyceride (mg/dL)	150	101.5	148.3	74.1	0.72	117.4	55.2	117.5	51.4	0.98
HDL-Cholesterol (mg/dL)	45.7	9.7	45.4	9.7	0.63	48.5	9.8	47.9	10.7	0.34
Waist circumference (cm)	81.4	6.5	81.4	6.8	0.85	77.8	8	77.8	7.6	0.99
Systolic blood pressure (mmHg)	117.5	15.1	118.6	15	0.19	114	16.1	113.6	16	0.62
Diastolic blood pressure (mmHg)	79.3	10.1	79.7	10.2	0.45	75.2	10	74.9	9.7	0.60
BMI (kg/m^2^)	23.6	2.6	23.6	2.6	0.91	24	2.9	24	2.6	0.94
Alcohol intake (g/day)	18	25.3	16.8	29.9	0.45	1.3	4.6	1.4	5.8	0.83
MET-h/week	170.6	103.7	159.3	97	0.06	159.5	100.2	154	89.9	0.32
Region (%)										
Ansung	581	39.2	131	36.9	0.43	640	44.9	143	41.6	0.26
Ansan	903	60.9	224	63.1		785	55.1	201	58.4	
Smoking (%)										
None	301	20.3	80	22.5	0.49	1377	96.6	334	97.1	0.87
Past	466	31.4	115	32.4		12	0.8	3	0.9	
Current	717	48.3	160	45.1		36	2.5	7	2.0	
Dietary fat composition										
Total fat (% of energy)	14.1	4.8	14.3	4.7	0.68	12.8	5.2	12.6	4.7	0.41
SFA (% of energy)	4.7	1.9	4.7	2	0.56	4.2	2.1	4.1	1.9	0.73
MUFA (% of energy)	3.8	1.7	3.9	1.7	0.41	3.4	1.8	3.3	1.6	0.33
PUFA (% of energy	2.3	0.8	2.3	0.8	0.27	2.2	0.8	2.2	0.9	0.53
Total fat (g/day)	33.3	17	34	17.1	0.54	28.4	16.9	27.8	14.1	0.52
SFA (g/day)	11	6.1	11.3	6.4	0.47	9.1	6.1	9.1	5.3	0.86
MUFA (g/day)	9	5.4	9.3	5.8	0.31	7.5	5.3	7.2	4.3	0.32
PUFA (g/day)	5.3	2.8	5.5	2.7	0.30	4.9	2.7	4.8	2.9	0.66

SD, standard deviation; HOMA-IR, homeostatic model assessment of insulin resistance; HDL, high-density lipoprotein; MET, metabolic equivalent task; SFA, saturated fatty acid; MUFA, monounsaturated fatty acid; PUFA, polyunsaturated fatty acid. ^1^ *p* Values based on *t*-tests for continuous variables and Chi-square tests for categorical variables.

**Table 3:** Sociodemographic, clinical, and lifestyle characteristics of the study participants at baseline according to the *CLOCK* rs1801260 genotype and dietary monounsaturated fatty acid (MUFA) intake.

**Men (*n* = 1839)**	**rs1801260 AA and MUFA ≤ 3.5% of Energy (*n* = 719)**		**rs1801260 AA and MUFA > 3.5% of Energy (*n* = 765)**		**rs1801260 AG + GG and MUFA ≤ 3.5% of Energy (*n* = 154)**		**rs1801260 AG + GG and MUFA > 3.5% of Energy (*n* = 201)**		
	**Mean**	**SD**	**Mean**	**SD**	**Mean**	**SD**	**Mean**	**SD**	***p* Value ^1^**
Age (years)	52.5 ^a^	8.7	49 ^b^	7.8	51.5 ^a^	8.6	49.4 ^b^	7.8	<0.0001
Insulin (µIU/mL)	6.6	4.5	6.6	3.7	6.5	2.9	6.4	3	0.87
Glucose (mg/dL)	86.5	17.2	87.9	17.2	87.1	19.9	88.2	19.7	0.4
HOMA-IR	1.4	1	1.4	0.8	1.4	0.7	1.4	0.7	0.97
Triglyceride (mg/dL)	154.1	113.1	146	89.1	154.4	81	143.6	68.1	0.29
HDL-Cholesterol (mg/dL)	44.9 ^b^	9.5	46.4 ^a^	9.7	46.1 ^ab^	10.7	44.8 ^b^	8.8	0.01
Waist circumference (cm)	81 ^b^	6.6	81.7 ^a^	6.5	81.2 ^ab^	6.7	81.6 ^ab^	6.9	0.21
Systolic blood pressure (mmHg)	118.7 ^a^	15.8	116.3 ^b^	14.3	120.3 ^a^	14.9	117.4 ^ab^	15	0.002
Diastolic blood pressure (mmHg)	79.4	9.8	79.1	10.4	80.1	10.3	79.4	10.2	0.71
BMI (kg/m^2^)	23.4 ^b^	2.6	23.9 ^a^	2.6	23.5 ^ab^	2.5	23.7 ^ab^	2.7	0.004
Alcohol intake (g/day)	14.8 ^b^	21.9	21.1 ^a^	27.8	19.4 ^a^	38.2	14.7 ^b^	21.5	<0.0001
MET-h/week	178.7 ^a^	106.4	163.1 ^bc^	100.6	154 ^bc^	98.5	163.4 ^ab^	95.8	0.005
**Women (*n* = 1769)**	**rs1801260 AA and MUFA ≤ 3.5% of energy (*n* = 696)**		**rs1801260 AA and MUFA > 3.5% of energy (*n* = 729)**		**rs1801260 AG + GG and MUFA ≤ 3.5% of energy (*n* = 168)**		**rs1801260 AG + GG and MUFA > 3.5% of energy (*n* = 176)**		
	**Mean**	**SD**	**Mean**	**SD**	**Mean**	**SD**	**Mean**	**SD**	***p* Value ^1^**
Age (years)	52.3 ^a^	8.6	47.8 ^b^	7.1	52.4 ^a^	8.7	47.2 ^b^	7.1	<0.0001
Insulin (µIU/mL)	7.4	4.8	7.2	4.2	7.8	3.7	7.4	3.3	0.58
Glucose (mg/dL)	82.1 ^a^	13.9	80.8 ^b^	7.9	81.4 ^ab^	8.8	83.1 ^a^	13.4	0.047
HOMA-IR	1.5	1	1.4	0.9	1.6	0.8	1.5	0.8	0.35
Triglyceride (mg/dL)	120.1	62.4	114.9	47.2	122.4	45.3	112.9	56.3	0.12
HDL-Cholesterol (mg/dL)	48.3	9.6	48.7	10	47.2	11.2	48.5	10.3	0.37
Waist circumference (cm)	78.9 ^ab^	8.3	76.7 ^c^	7.5	79.1 ^a^	7.6	76.5 ^c^	7.4	<0.0001
Systolic blood pressure (mmHg)	115.9 ^a^	16.9	112.3 ^b^	15.1	115.5 ^a^	16.6	111.7 ^b^	15.1	<0.0001
Diastolic blood pressure (mmHg)	76.2 ^a^	10	74.3 ^b^	10	75.4 ^ab^	9.7	74.4 ^ab^	9.7	0.003
BMI (kg/m^2^)	24	3	23.9	2.7	24.1	2.5	23.8	2.8	0.77
Alcohol intake (g/day)	0.8 ^c^	2.6	1.8 ^a^	5.8	1 ^b^	3.6	1.7 ^ab^	7.2	0.0002
MET-h/week	172.4 ^a^	110.4	147.3 ^b^	87.7	168.8 ^a^	97.9	139.8 ^b^	79.2	<0.0001

HOMA-IR, homeostatic model assessment of insulin resistance; MET, metabolic equivalent task; SD, standard deviation. ^1^ *p* Value based on one-way ANOVA test. ^abc^ Bonferroni post hoc test: means with the same letter indicate no significant difference. Any difference between two means carrying different letters is significant (*p* Value < 0.05).

**Table 4:** Hazard ratios (HR) ^1^ for metabolic syndrome depending on the *CLOCK* rs1801260 genotype.

	Person-Years	Cases/Total	HR	(95% CI)		*p* Value
**Men (*n* = 1839)**						
rs1801260						
AG + GG	3463	178/355	1.18	(1.00–1.40)		0.047
AA	15,069	671/1484	1.00			
**Women (*n* = 1769)**						
rs1801260						
AG + GG	3553	163/344	1.13	(0.96–1.35)		0.15
AA	14,991	646/1425	1.00			

HR, hazard ratio; CI, confidence interval. ^1^ Adjusted for age (in years, continuous), region (Ansung, Ansan), smoking (none, past, current), alcohol intake (g/day), metabolic equivalent (MET)-h/week (continuous), family history of type 2 diabetes (yes/no), and BMI (in kg/m^2^, continuous).

**Table 5:** Hazard ratios (HR) ^1^ for metabolic syndrome depending on the *CLOCK* rs1801260 genotype and dietary monounsaturated fatty acid (MUFA) intake.

	Person-Years	Cases/Total	HR	(95% CI)	*p* Value	*p* Interaction
**Men (*n* = 1839)**						
AA and MUFA ≤ 3.5% of energy	7175	327/719	1.07	(0.91–1.25)	0.40	0.24
AA and MUFA > 3.5% of energy	7894	344/765	1.00			
AG + GG and MUFA ≤ 3.5% of energy	1405	84/154	1.42	(1.12–1.81)	0.004	
AG + GG and MUFA > 3.5% of energy	2058	94/201	1.09	(0.86–1.37)	0.48	
**Women (*n* = 1769)**						
AA and MUFA ≤ 3% of energy	6984	357/696	1.01	(0.86–1.20)	0.86	0.96
AA and MUFA > 3% of energy	8007	289/729	1.00			
AG + GG and MUFA ≤ 3% of energy	1625	90/168	1.16	(0.91–1.47)	0.24	
AG + GG and MUFA > 3% of energy	1928	73/176	1.13	(0.87–1.46)	0.36	

HR, hazard ratio; CI, confidence interval. ^1^ Adjusted for age (in years, continuous), region (Ansung, Ansan), smoking (none, past, current), alcohol intake (g/day), metabolic equivalent (MET)-h/week (continuous), family history of type 2 diabetes (yes/no), and BMI (in kg/m^2^, continuous).

## Data Availability

The dataset used in this study (Ansan-Ansung Cohort Study of the KoGES) can be provided after review and evaluation of research plan by the Korea National Institute of Health, Korea Centers for Disease Control and Prevention (http://nih.go.kr/contents.es?mid=a50401010400).
